# Optical properties of GaP/GaNP core/shell nanowires: a temperature-dependent study

**DOI:** 10.1186/1556-276X-8-239

**Published:** 2013-05-16

**Authors:** Alexander Dobrovolsky, Shula Chen, Yanjin Kuang, Supanee Sukrittanon, Charles W Tu, Weimin M Chen, Irina A Buyanova

**Affiliations:** 1 Department of Physics, Chemistry and Biology, Linköping University, Linköping, 581 83, Sweden; 2 Department of Physics, University of California, La Jolla, San Diego, California, 92093, USA; 3 Graduate Program of Material Science and Engineering, University of California, La Jolla, San Diego, California, 92093, USA; 4 Department of Electrical and Computer Engineering, University of California, La Jolla, San Diego, California, 92093, USA

**Keywords:** Nanowires, III-V semiconductors, Photoluminescence, 68.65.La, 78.55.Cr, 61.72.-y

## Abstract

Recombination processes in GaP/GaNP core/shell nanowires (NWs) grown on Si are studied by employing temperature-dependent continuous wave and time-resolved photoluminescence (PL) spectroscopies. The NWs exhibit bright PL emissions due to radiative carrier recombination in the GaNP shell. Though the radiative efficiency of the NWs is found to decrease with increasing temperature, the PL emission remains intense even at room temperature. Two thermal quenching processes of the PL emission are found to be responsible for the degradation of the PL intensity at elevated temperatures: (a) thermal activation of the localized excitons from the N-related localized states and (b) activation of a competing non-radiative recombination (NRR) process. The activation energy of the latter process is determined as being around 180 meV. NRR is also found to cause a significant decrease of carrier lifetime.

## Background

GaNP has recently attracted much attention as a promising material for applications in optoelectronic and photonic devices, such as light-emitting diodes [1-3]. The incorporation of N in GaP allows one to tune the band gap energy and also to change the band gap character from an indirect one in GaP to a direct-like one in the GaNP alloys, leading to improvements in light emission efficiency [2,3]. A small lattice mismatch of GaNP to Si also provides a unique opportunity to combine high optical efficiency of the III-V compound semiconductors with the capabilities of mature silicon technologies [4-6]. Unfortunately, the properties desired for optoelectronic applications have not been fully utilized due to the degradation of optical quality of GaNP caused by the formation of defects that act as centers of non-radiative recombination (NRR) [7]. The NRR processes often dominate carrier recombination and are largely responsible for a reduced optical efficiency of optoelectronic devices [8].

The growth of semiconductor materials in the form of nanostructures, such as nanowires (NWs), often allows suppression of defect formation and therefore offers a possibility to overcome the limitation imposed by NRR that is inherent to higher dimensional layers/structures. It also provides increased flexibility in structural design, thanks to confinement effects. In fact III-V NWs are currently considered as being among the key material systems for future optoelectronic and photonic devices integrated with Si [9-11]. Recently, the epitaxial growth of GaP/GaNP core/shell NWs on Si (111) has been reported [12]. High optical quality of these structures has been demonstrated based on the observation of intense photoluminescence (PL) emission from a single NW [13]. In spite of the high optical quality, fast PL decay caused by NRR processes in the NWs has been reported. The purpose of this work is to gain a better understanding on the quenching processes of the PL intensity from GaP/GaNP core/shell NWs based on temperature-dependent studies by continuous wave (cw) and also time-resolved PL spectroscopies.

## Methods

**Figure 1:**
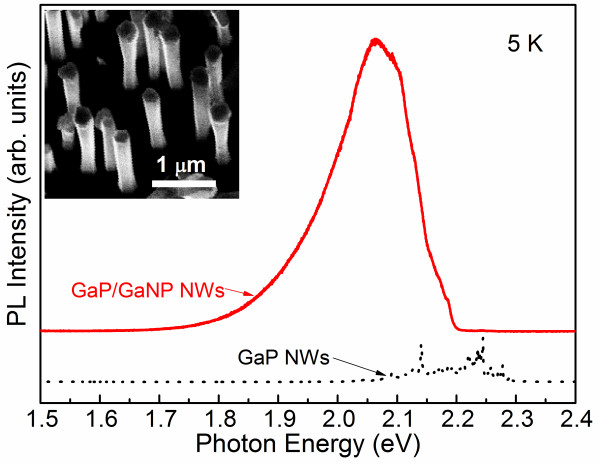
**PL spectra from the studied NWs.** The inset: an SEM image of the GaP/GaNP NWs.

The GaP and GaP/GaNP NW samples were grown by gas source molecular beam epitaxy (MBE) on (111)-oriented Si substrates [12]. Scanning electron microscopy (SEM) showed that NWs are hexagonal in shape (inset in Figure 1), indicating that NWs were epitaxially grown following the Si [111] crystal orientation. The NWs are uniform in sizes and have an axial length of about 2.5 μm, a total diameter of about 220 nm for the GaP/GaNP NWs, and a typical diameter of approximately 110 nm for the GaP NWs. The N content in the GaNP NW shell was estimated [12] to be approximately 0.9% on average from room-temperature (RT) PL data. For a comparison, a 750-nm-thick GaN_0.009_P_0.991_ epilayer grown by gas-source MBE on a (001)-oriented GaP substrate was also investigated. PL measurements were carried out in a variable temperature cryostat under optical excitation by the 325-nm line of He-Cd laser, the 532-nm line of a solid state laser or the 633-nm line of a He-Ne laser. The resulting PL was detected by a liquid nitrogen cooled charge coupled device after passing through a grating monochromator. Time-resolved PL was excited by a pulsed Ti/sapphire picosecond laser with a photon wavelength of 375 nm and a pulse repetition frequency of 76 MHz and was detected using a streak camera system.

## Results and discussion

Figure 1 shows representative PL spectra measured from the GaP NW (the dotted line, black online) and the GaP/GaNP core/shell NW samples (the solid line, red online) at 5 K using the 325-nm line of a solid state laser as an excitation source. The PL emission from the GaP NW is rather weak and is dominated by a series of relatively sharp lines within the 2.05 to 2.32 eV spectral range due to the recombination of excitons bound to various residual impurities. Some of the PL lines are very similar to the previously reported emissions due to the recombination of excitons bound to isoelectronic centers involving N impurity, e.g., from an isoelectronic B_Ga_-N_P_ center and its phonon replica [14]. Though the studied GaP NWs are intentionally undoped, the formation of the N-related centers may be caused by contamination of the growth chamber. Further studies aiming to clarify the exact origin of these emissions are currently in progress.

The PL spectra are significantly modified in the GaP/GaNP core/shell NW. First of all, the sharp excitonic lines are replaced by a broad PL band with a rather asymmetric lineshape that peaks at around 2.06 eV (Figure 1). This emission originates from radiative recombination of excitons trapped at various N-related localized states [13] in the GaNP shell. Secondly, a significant increase of the integrated PL intensity (by about 20 times) is observed which is largely related to the N-induced transition from the indirect bandgap in GaP to a direct bandgap in the GaNP alloy [3]. The observed high efficiency of the radiative recombination in the GaP/GaNP core/shell NW implies that this material system could be potentially promising for applications as efficient nano-sized light emitters.

**Figure 2:**
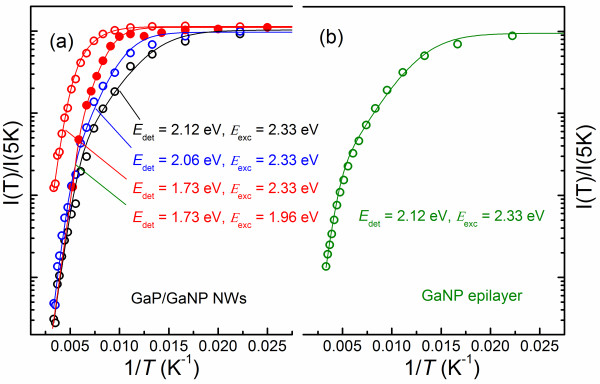
Arrhenius plots of the PL intensity measured at different detection energies from the GaP/GaNP NWs (a) and GaNP epilayer (b).

For practical device applications, it is essential that the high efficiency of radiative recombination is sustained up to RT. Therefore, recombination processes in the studied structures were further examined by employing temperature-dependent PL measurements. In the case of GaP NWs, temperature increase was found to cause a dramatic quenching of the PL intensity so that it falls below the detection limit of the measurement system at measurement temperatures *T* exceeding 150 K. For the GaP/GaNP NW, on the other hand, the PL emission was found to be rather intense at RT even from an individual NW, though significantly weaker than that at 5 K. Moreover, thermal quenching is found to be more severe for the high energy PL components which lead to an apparent red shift of the PL maximum position at high *T*. To get further insights into the mechanisms responsible for the observed thermal quenching, we have analyzed Arrhenius plots of the PL intensity at different detection energies (*E*_det_) as shown in Figure 2a. The analysis was performed for constant detection energies since (a) the temperature-induced shift of the bandgap energy is significantly suppressed in GaNP alloys [15], and (b) spectral positions of the excitons bound to various deep-level N-related centers do not one-to-one follow the temperature-induced shift of the bandgap energy. This approximation defines error bars of the deduced values as specified below. All experimental data (shown by the symbols in Figure 2) can be fitted bywhere *I*(*T*) is the temperature-dependent PL intensity, *I*(0) is its value at 4 K, *E*_1_ and *E*_2_ are the activation energies for two different thermal quenching processes, and *k* is the Boltzman constant (the results of the fitting are shown by the solid lines in Figure 2a). The first activation process that occurs within the 30 to 100 K temperature range is characterized by the activation energy *E*_1_ ranging between 40 (at *E*_det_ = 2.17 eV) and 60 meV (at *E*_det_ = 2.06 eV). The contribution of this process is most pronounced for high energy PL components that correspond to the radiative recombination at the N-related localized states with their energy levels close to the GaNP band edge. The quenching of the high energy PL components is accompanied by a slight increase in the PL intensity at low *E*_det_. Therefore, this process can be attributed to the thermal ionization of the N-related localized states. Such ionization is expected to start from the N-states that are shallower in energy. The thermally activated excitons can then be recaptured by the deeper N states, consistent with our experimental observations. We note that the determined values of *E*_1_ do not one-to-one correspond to the ‘apparent’ depth of the involved localized states deduced simply from the distance between *E*_det_ and the bandgap energy of the GaNP. This is, however, not surprising since such correspondence is only expected for the no-phonon excitonic transitions whereas recombination of excitons at strongly localized states (such as the monitored N states) is usually dominated by phonon-assisted transitions due to strong coupling with phonons.

(1) $$I\left(T\right)=\frac{I\left(0\right)}{1+C_{1}e^{-E_{1}/\mathrm{kT}}+C_{2}e^{-E_{2}/\mathrm{kT}}}$$

The second thermal quenching process is characterized by the activation energy *E*_2_ of approximately 180 ± 20 meV, which is the same for all detection energies. This process becomes dominant at *T* > 100 K and leads to an overall quenching of the PL intensity irrespective of detection energies. We therefore ascribe it to thermal activation of competing non-radiative recombination which depletes photo-created free carriers and, consequently, causes a decrease in the PL intensity. It is interesting to note that the competing NRR process remains active even when the excitation photon energy (*E*_exc_) is tuned to 1.96 eV, which is below the GaNP bandgap. Indeed, Arrenius plots of the PL intensity measured at *E*_det_ = 1.73 eV under *E*_exc_ = 2.33 eV (the open circles in Figure 2a) and *E*_exc_ = 1.96 eV (the dots in Figure 2a), i.e., under above and below bandgap excitation, respectively, yield the same activation energy *E*_2_. In addition, the PL thermal quenching under below bandgap excitation seems to be even more severe than that recorded under above bandgap excitation. At first glance, this is somewhat surprising as the 1.96 eV photons could not directly create free electron–hole pairs and will be absorbed at N-related localized states. However, fast thermal activation of the photo-created carriers from these localized states to band states will again lead to their capture by the NRR centers and therefore quenching of the PL intensity. Moreover, the contribution of the NRR processes is known to decrease at high densities of the photo-created carriers due to partial saturation of the NRR centers which results in a shift of the onset of the PL thermal quenching to higher temperatures. In our case, such regime is likely realized for the above bandgap excitation. This is because of (a) significantly (about 1,000 times) lower excitation power used under below bandgap excitation (restricted by the available excitation source) and (b) a high absorption coefficient for the band-to-band transitions.

The revealed non-radiative recombination processes may occur at surfaces, the GaNP/GaP interface or within bulk regions of GaNP shell. The former two processes are expected to be enhanced in low-dimensional structures with a high surface-to-volume ratio whereas the last process will likely dominate in bulk (or epilayer) samples. Therefore, to further evaluate the origin of the revealed NRR in the studied NW structures, we also investigated the thermal behavior of the PL emission from a reference GaNP epilayer. It is found that thermal quenching of the PL emission in the epilayer can be modeled, within the experimental accuracy, by the same activation energies as those deduced for the NW structure. This is obvious from Figure 2b where an Arrhenius plot of the PL intensity measured at *E*_det_ = 2.12 eV under *E*_exc_ = 2.33 eV from the epilayer is shown. However, the contribution of the second activation process (defined by the pre-factor *C*_2_ in Equation 1) is found to be larger in the case of the GaNP/GaP NWs. This suggests that the formation of the responsible defects is facilitated in the lower dimensional NWs and that the defects could be at least partly located either at the surface of the GaNP shell or at the GaNP/GaP hetero-interface, consistent with the results of [13].

**Figure 3:**
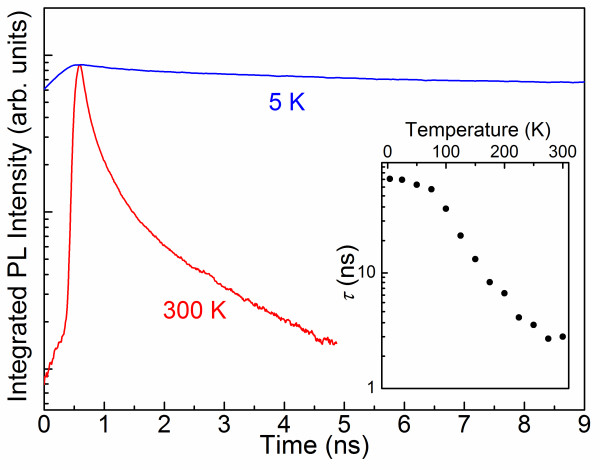
Decays of the integrated PL intensity measured from the GaP/GaNP NWs at 5 K and RT.

The activation of the NRR recombination processes at elevated temperatures is also confirmed by the performed time-resolved PL measurements. Typical decay curves of the integrated PL intensity at 5 K and RT are shown in Figure 3. At 5 K, the PL decay is found to be rather slow, i.e., with the decay time *τ* of the dominant decay component longer than 60 ns (the exact value of *τ* could not be determined from the available data due to the high repetition frequency of the laser pulses). Such slow decay is likely dominated by the radiative lifetime *τ*_r_ as it is of the same order of magnitude as previously determined for the radiative transitions within the N-related localized states in the GaNP epilayers [3]. A temperature increase above 100 K causes significant shortening of the PL decay, down to several ns at RT (see the inset in Figure 3). The measured decay time contains contributions from both radiative and NRR processes so that $\frac{1}{\tau }=\frac{1}{\tau_{r}}+\frac{1}{\tau_{\mathrm{nr}}}$ where τ_nr_ denotes the non-radiative decay time. Therefore, the observed dramatic shortening of the measured decay time at elevated temperature implies thermal activation of non-radiative carrier recombination, consistent with the results of cw-PL measurements (Figure 2).

## Conclusions

In summary, we have investigated the recombination processes in the GaP NW and GaP/GaNP core/shell NW structures grown on a Si substrate using temperature-dependent cw and time-resolved PL spectroscopies. The GaP/GaNP core/shell NWs are concluded to be a potentially promising material system for applications as efficient nano-sized light emitters that can be integrated with Si. However, the efficiency of radiative recombination in the NWs is found to degrade at elevated temperatures due to the activation of the competing NRR process that also causes shortening of the PL decay time. The thermal activation energy of the NRR process is determined as being around 180 meV.

## Competing interests

The authors declare that they have no competing interests.

## Authors' contributions

AD carried out the experiments and analyzed the data with guidance from IAB and WMC. YK and SS performed the growth of the NWs with guidance from CWT. IAB wrote the final version of the manuscript with contributions from the co-authors. All authors read and approved the final manuscript.
